# Contemporary connectivity is sustained by wind- and current-driven seed dispersal among seagrass meadows

**DOI:** 10.1186/s40462-015-0034-9

**Published:** 2015-04-02

**Authors:** Leonardo Ruiz-Montoya, Ryan J Lowe, Gary A Kendrick

**Affiliations:** The School of Earth and Environment, The University of Western Australia, Crawley, Western Australia Australia; The School of Plant Biology, The University of Western Australia, Crawley, Western Australia Australia; The University of Western Australia Oceans Institute, Crawley, Western Australia Australia; ARC Centre of Excellence for Coral Reef Studies, Crawley, Western Australia Australia

**Keywords:** Coastal circulation, Dispersal, Population connectivity, *Posidonia australis*, Seagrasses

## Abstract

**Background:**

Seagrasses are clonal marine plants that form important biotic habitats in many tropical and temperate coastal ecosystems. While there is a reasonable understanding of the dynamics of asexual (vegetative) growth in seagrasses, sexual reproduction and the dispersal pathways of the seeds remain poorly studied. Here we address the potential for a predominantly clonal seagrass, *P. australis*, to disperse over long distances by movement of floating fruit via wind and surface currents within the coastal waters of Perth, Western Australia. We first simulated the dominant atmospheric and ocean forcing conditions that are known to disperse these seagrass seeds using a three-dimensional numerical ocean circulation model. Field observations obtained at 8 sites across the study area were used to validate the model performance over ~2 months in summer when buoyant *P. australis* fruit are released into the water column. *P. australis* fruit dispersal trajectories were then quantified throughout the region by incorporating key physical properties of the fruit within the transport model. The time taken for the floating fruit to release their seed (dehiscence) was incorporated into the model based on laboratory measurements, and was used to predict the settlement probability distributions across the model domain.

**Results:**

The results revealed that high rates of local and regional demographic connectivity among *P. australis* meadows are achieved via contemporary seed dispersal. Dispersal of seeds via floating fruit has the potential to regularly connect meadows at distances of 10s of kilometres (50% of seeds produced) and infrequently for meadows at distances 100 s km (3% of seeds produced).

**Conclusions:**

The spatial patterns of seed dispersal were heavily influenced by atmospheric and oceanographic conditions, which generally drove a northward pattern of connectivity on a regional scale, but with geographical barriers influencing finer-scale connectivity pathways at some locations. Such levels of seed dispersal infer greater levels of ecological and genetic connectivity and suggest that seagrasses are not just strongly clonal.

**Electronic supplementary material:**

The online version of this article (doi:10.1186/s40462-015-0034-9) contains supplementary material, which is available to authorized users.

## Background

Quantifying population connectivity within coastal ecosystems is a crucial component of the management and conservation of many marine populations, especially when it becomes necessary to forecast how increasing environmental pressures such as water quality degradation, species invasions and climate change will impact these ecosystems [1]. In order to accurately assess marine connectivity, it is imperative to understand the dominant physical transport processes in a region (e.g., tides, waves, wind, etc.) and how the biological dispersal capabilities of different species interact with these physical dynamics. It is ultimately these biophysical interactions that determine how the spatial connectivity pathways of marine populations are influenced over a broad range of spatial scales, depending on transport mechanisms that are present, as well as the physical characteristics of the propagule that is being dispersed [2-4].

Seagrasses are marine plants with the ability to reproduce both asexually (clonally) and sexually (via seeds). There is a reasonable understanding of the dynamics of asexual seagrass reproduction that has led to the development of meadow expansion models based on rates of linear growth [5,6], nonlinear models of seagrass growth [7,8] and even three-dimensional (3D) models of structural formation of meadows (e.g. [9]). Conversely, sexual reproduction, seed dispersal and recruitment in seagrasses remain much more poorly studied [10]. Seed dispersal is the process governed by the movement from the initial release of a fruit by the parent plant to the time when the seed settles to a location where it may recruit. This trajectory is affected by different physical and biological components (see Levin et al. [11] for a general review of seed dispersal). In the coastal and estuarine environments that seagrasses inhabit, flow generated by currents and waves generate bed shear stresses capable of transporting seeds in the bottom boundary layer [4,12,13]. However, the positively buoyant fruit of some seagrass species are transported at the air-water interface by surface ocean currents as well as direct wind forces, which can provide a mechanism for long distance dispersal [10,14-16]. Ultimately these seeds must also settle in favourable substrata and in suitable environmental conditions for recruitment to be successful [15].

For seagrasses, most attempts to quantify dispersal distances have tended to be only very crude estimates, e.g., as derived from rough (order-of-magnitude) measures of background ocean currents and seed lifecycle characteristics, or inferred from genetics [17]. Kendrick et al. [10] emphasised the wide ranges of dispersal distances that have been reported for different seagrass species. Dispersal distance estimates vary from only a few meters for the negatively buoyant seeds of *Zostera marina* when on the sediment surface [13], to hundreds of kilometres in studies of the fruit of *Thalassia* obtained by a genetic metapopulation study [18] and estimates of surface travel of *Enhalus* and *Thalassia* fruit by extreme events (e.g., typhoons) [15,16]. Despite the importance of dispersal to demographic connectivity in seagrasses, there are still major gaps in our understanding of the spatial implications of the connectivity of distant populations and the importance of locally- versus regionally-derived recruitment processes on individual populations [17]. To develop a predictive understanding of demographic connectivity in seagrasses, we thus need to know: 1) seed production estimates and the rate at which these propagules are released from the parent plant, 2) the physical vector responsible for dispersal or where these seeds are transported to and over what time scale, and 3) the survival rates of seeds once they settle. We can estimate seed production (e.g. [19,20]), investigate germination and survival rates under controlled conditions (e.g. [21-23]) and sometimes even observe natural recruitment [15,24,25]. However, for the most part we still do not know where seeds are ultimately transported to in most seagrass ecosystems, and hence where new recruits that may structure seagrass populations originate from.

The use of process-based models that incorporate both predictions of the key hydrodynamic transport mechanisms as well as the physical characteristics of seeds and fruit have the capability to advance our understanding of dispersal pathways in complex coastal systems [1]. This approach has only been used for seagrasses in a very limited number of studies, focusing on dispersal of the European populations of *Zostera marina*. Källström et al., [26] empirically estimated a maximum dispersal distance of ~150 km from wind fields acting on rafting shoots bearing seeds. However, wind was the only forcing mechanism considered in the model and hence no hydrodynamic information was incorporated. Erftemeijer et al., [27] used a 3D ocean model to simulate the trajectories of *Z. marina* shoots released inside a large estuary and predicted dispersal distances of up to 130 km over a 3–4 week period. However, transport was due to the surface currents but there was no data to accurately account for additional transport from windage. In general, this class of particle tracking modelling has proven to be successful for predicting the transport of seagrass shoots, fish larvae (e.g. [28,29]) and corals [30], although the accuracy is dependent on how well the properties of the dispersing propagule are known. Ruiz-Montoya et al., [4] have already described how dispersal propagules of *P. australis* move under different wind and current forcing, forming the basis for parameterizing our modelling of seed dispersal in this study.

The southwest region of Australia has one of the highest diversities of temperate seagrasses in the world throughout a 2500 km coastline [31]. The dominant genera in the region are *Posidonia* and *Amphibolis*, and they create large mono-specific meadows with smaller species as understorey [31,32]. The fruit of *P. australis* are released during the austral summer (November-December), and because these fruit are less dense than seawater, they rapidly float to the water surface where they are transported by ocean surface currents and wind drag (‘windage’) acting on their air exposed surface. This flotation period lasts until dehiscence (seed release) occurs, which can take up to ~5 days [4]. After dehiscence, the negatively buoyant seed settles at ~10 cm s^−1^ and once it reaches the seafloor, requires shear stresses greater than ~100 mPa to be moved. This energy is not likely to be reached by unidirectional currents in the region (e.g. due to wind and tide), but oscillatory wave-driven flows may further mobilize the seeds over short distances, especially during storm conditions [4].

**Figure 1:**
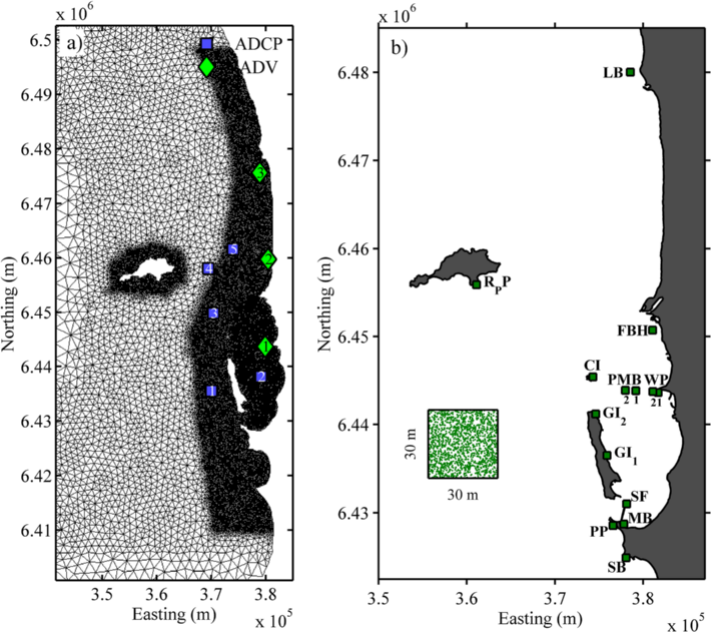
**Study area showing a) the unstructured model grid with increasing resolution in the shallow coastal areas and b) Seagrass meadow locations representing both fruit release sites and potential settlement areas.** The green dots represent how the release was random within the cell. The instruments used were: ADV which stands for Acoustic Doppler Velocimeter and ADCP for Acoustic Doppler Current Profiler.

The Perth coastal area is a relatively shallow environment (~20 m) with some islands and several rocky reefs running parallel to the coast (Figure 1). The region experiences a diurnal tidal regime with a microtidal range of only ~0.6 m. The offshore (shelf) waters are dominantly forced by an alongshore pressure gradient that produces a southward flow known as the Leeuwin Current (LC). The presence of the LC shifts the tropical bioregion along Western Australia south, and despite some weakening of its strength in summer, it is often significant year round [33,34]. Although the Leeuwin current has a strong influence on the circulation of the shelf (i.e., depths >100 m), Ruiz-Montoya and Lowe [35] found that the inshore coastal circulation was opposite (i.e., dominantly northward) throughout the summer period, which was driven by the strong northward winds present that also kept the water column in the coastal region well-mixed during this period.

In this study we hypothesize that *Posidonia australis* populations throughout the south-western margin of Australia have a potential for high contemporary connectivity over large distances due to their floating fruit. We investigate this potential connectivity by modelling the two-dimensional dispersal patterns of *P. australis* fruit in the coastal waters of Perth, Western Australia, driven by a combination of transport by modelled ocean surface currents as well as direct windage.

## Results

### Hydrodynamic model performance

**Figure 2:**
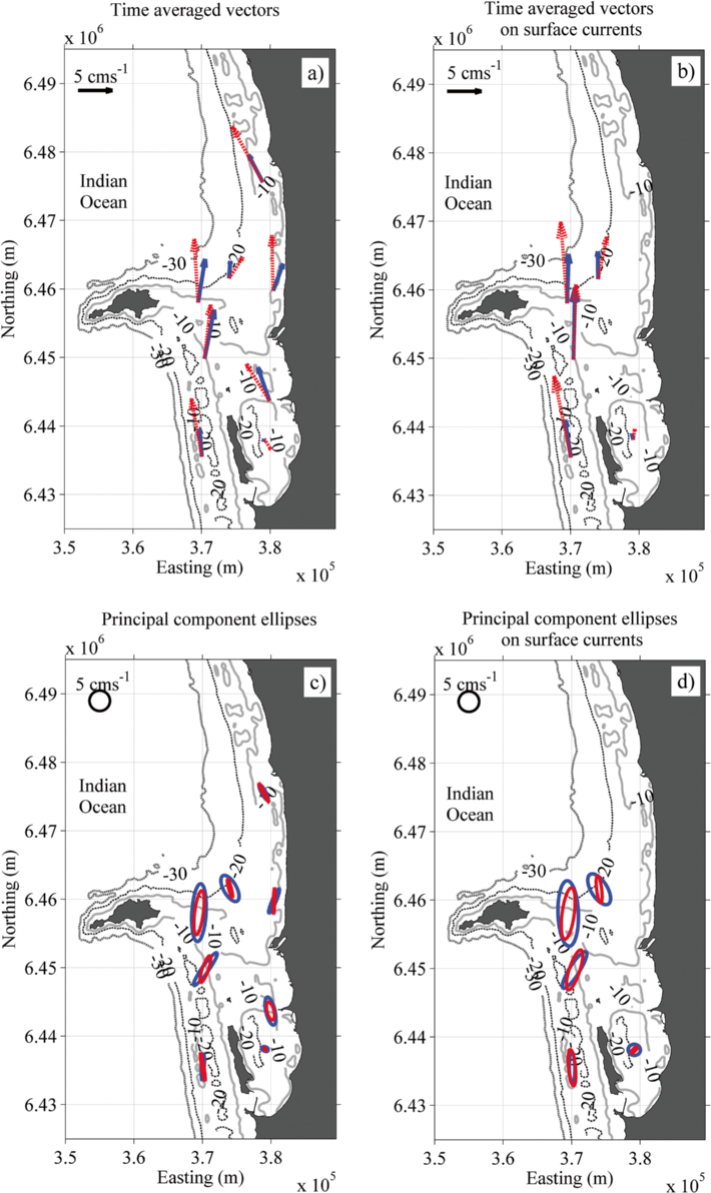
**Comparison of: a) the modelled (red) and observed (blue) depth-averaged current vectors averaged over the ~2 month experiment period; b) the surface currents at the deeper sties; c) the depth-averaged current variance ellipses; and d) the surface current variance ellipses with radii representing one standard deviation of the flow.**

Overall, the 3D hydrodynamic model provided robust predictions of the dominant transport processes throughout the study region (Figure 2). The current and water level time series were quantitatively compared with the field observations at all 8 sites during the 2 month hindcast experiment period. The experiment-averaged current vectors predicted by the model (both depth-averaged and surface) generally showed good agreement with the field observations (Figure 2a,b). Both the field observations and model predictions reveal that the relatively consistent northward winds during this summer study period drove a mean northward flow in the coastal waters off Perth. At some locations the model slightly overpredicted this northward transport (Figure 2a,b). This discrepancy is most evident at sites P1, P4 and V3.

At site P2 in the semi-enclosed embayment of Cockburn Sound, the depth-averaged flow is relatively weak (<0.02 m s^−1^); hence resulting in a very weak depth-averaged signal. However, the surface currents were much more accurately predicted (Table 1). The modelled current variance ellipses are also generally in very good agreement with the observations, both in terms of their magnitudes and orientations, including for both the depth-averaged and surface currents (Figure 2c,d). The orientations of these ellipses are strongly influenced by the local bathymetry of the sites.

**Table 1:** **Model** ***Skill*** **computed via Eq. (** 1 **) for the subtidal depth-averaged currents, subtidal surface currents and water levels**

**Site**	**Depth- averaged (east)**	**Depth- averaged (north)**	**Depth- averaged speed**	**Surface (east)**	**Surface (north)**	**Surface speed**	**Water level**
P1	0.19	0.79	0.58	0.41	0.76	0.51	0.95
P2	0.44	0.37	0.28	0.64	0.62	0.30	0.93
V1	0.75	0.85	0.76	-----	-----	-----	-----
P3	0.59	0.87	0.74	0.35	0.79	0.75	0.96
P4	0.76	0.80	0.67	0.81	0.78	0.61	0.96
V2	0.42	0.81	0.74	-----	-----	-----	0.95
P5	0.42	0.78	0.60	0.53	0.69	0.53	0.95
V3	0.64	0.76	0.65	-----	-----	-----	0.94

A value of 1 represents perfect agreement while a value of zero, total disagreement.

**Figure 3:**
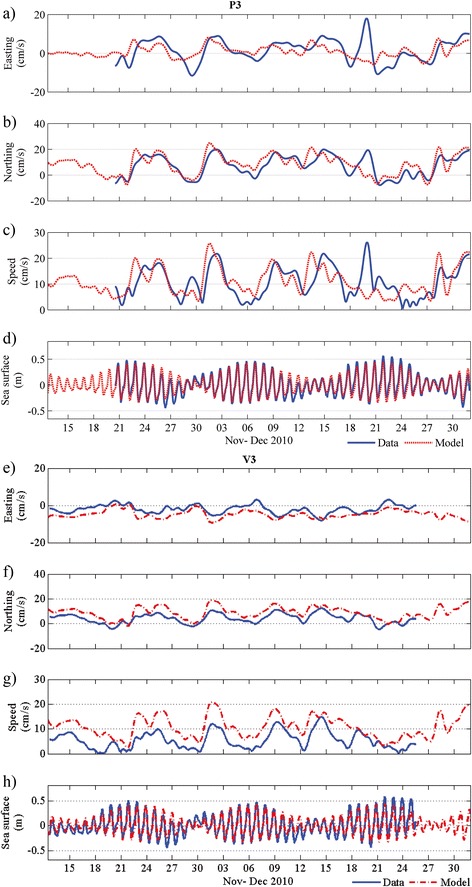
**Comparison between measured and modelled subtidal depth averaged velocities at site P3 (a,b,c) and V3 (e,f,g).** A comparison of the measured versus modelled water levels at the same two sites **(d,h).**

Water level variability across the study domain was reproduced by the model with very high Skill, i.e. averaging 0.95 among the sites (Table 1; Figure 3d,h). Both the depth-averaged and surface subtidal velocity components were also generally well predicted by the model at all sites, especially for the most dominant north–south velocity component (~0.75), which at most sites roughly coincides with the major axis of the current variance (see Table 1). Figure 3 shows a detailed time series comparison of the field observations and model results of the currents at two representative sites, including: an offshore site P3 at the edge of the Perth lagoon and a nearshore site at V3. For most of the period there was good model agreement, except for a period around the 20 of December when there is a relatively large discrepancy. As detailed in Ruiz-Montoya and Lowe [35], during this time a large coastally-trapped wave train generated by a tropical low ~1000 km north had a substantial influence on the circulation of this coastal region; this transient offshore forcing is not included in the model. Nevertheless, seed dispersal simulations detailed below were only conducted during the most likely release period (thus ending on 15 Dec) so this event would have no influence on the dispersal results.

### Dispersal results

**Figure 4:**
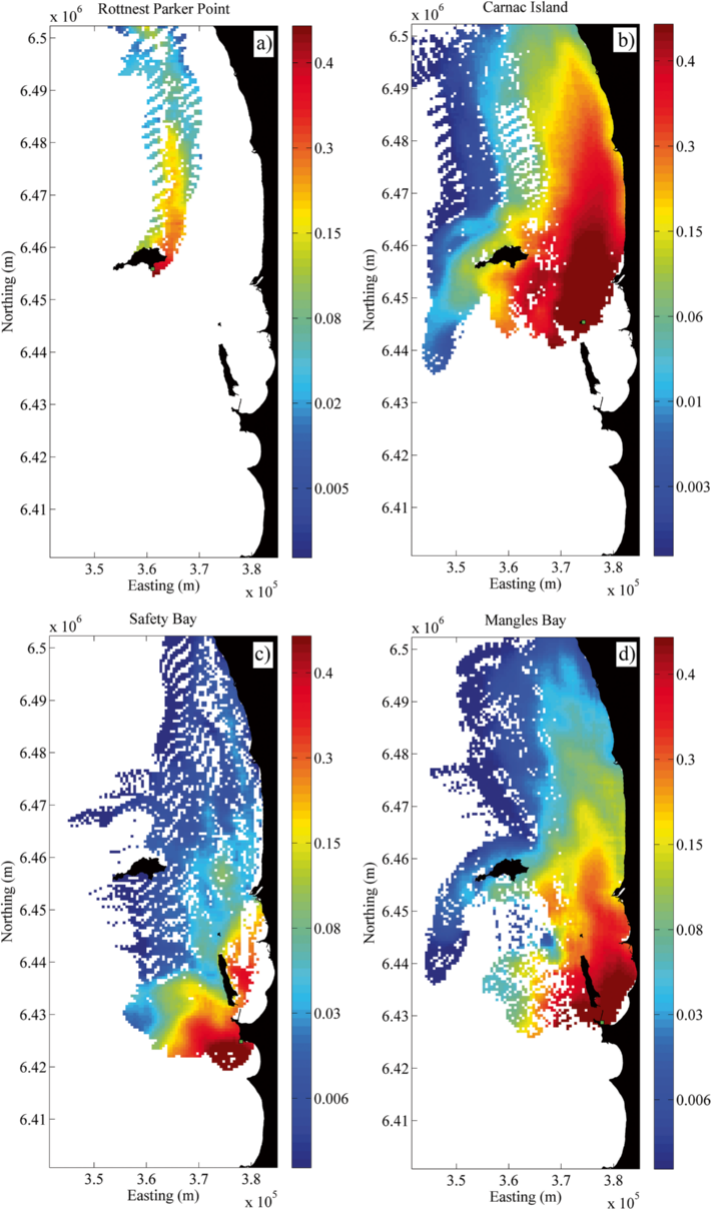
**Maps showing settlement probability locations for different sources. a)** Offshore site at Rottnest Island (R_PP) sheltered on the west and north with very high loss of fruit, **b)** Exposed site at Carnac Island (CI) over a deeper meadow (~10 m), allowing greater flows to carry fruit away with high probabilities of dehiscence over larger areas. **c)** Coastal site within Safety Bay (SB), where local circulation transported the fruit rapidly offshore, thus facilitating LDD. **d)** For this coastal sheltered site (MB) in the Cockburn embayment wind surface currents also allowed for broad dispersal with limited interruptions to the northward flow.

**Figure 5:**
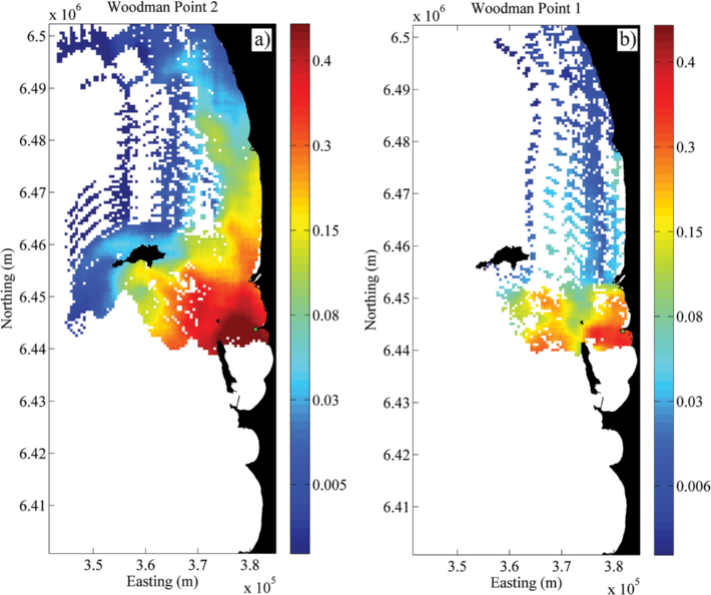
**Comparison of probability of settlement at two adjacent sites near Woodman Point separated by only ~500 m; a) western facing location (WP1) with significant export due to westward winds driving fruit offshore into exposed waters, b) southward facing site (WP2) with high loss of fruit stranded on the beach and only occasional export (thereby acting as a sink).**

Northward transport of seagrass fruit reached ~90 km whilst the maximum southward transport was only ~5 km (Figure 4). In the cross-shore (east–west) direction, the fruit could be transported ~40 km offshore. Combining the alongshore (~90 km) and cross-shore (~40 km) transport distances suggests a potential dispersal shadow of ~4000 km^2^ at offshore release sites. For inshore areas, where release sites are sheltered and flow decreases, the dispersal shadow is halved (~2000 km^2^). These dispersal areas were restricted by the size of our domain, as some particles were lost out of the domain through the northern boundary. In these simulations there were also some notable responses to local (fine-scale) water circulation patterns. For example, many seeds were lost from the domain at sites such as Rottnest (R_PP) (Figure 4a) and WP1 (Figure 5b), resulting in a relatively low probability of settlement inside the domain. In contrast, WP2, which is only ~500 m away from WP1, presents a much broader area of high probability settlement due to its orientation with land facing to the east. Safety Bay (SB, Figure 4c) is adjacent to land at the north; however the water movement induced by the open embayment allowed some fruit to be exported with some southward transport. Overall, although some fruit were capable of travelling long distances, the majority (~60%) of the fruit were predicted to dehisce within the first 20 km or less, given that dehiscence most likely occurred during the first couple of days.

The particles reached distances of ~90 km during the 5 day period; however, a fraction left the domain before this time so larger dispersal distances would also be possible (this is discussed further below). Model runs without the extra windage significantly reduced these transport distances by ~15 km on average, but the response varied among sites (Table 2). At many of the nearshore release sites, fruit were transported to shore by dominant winds blowing from the southwest, especially in the afternoon due to the strong and regular sea breeze cycle in this region. This is apparent by the smaller median distances observed from sites close to shore (SB, WP1) when windage was included in the model (Table 2). However, a large quantity of seeds also travelled to the west (i.e., towards Rottnest Island) due to the strong winds blowing from the east (mainland) that tend to prevail in the early morning during summer.

**Table 2:** **Statistics of the dispersal distances predicted from the model with (left) and without windage (right)**

**Windage**					**No Windage**			
			**Percentile**	**Left**			**Percentile**	**Left**
	**Mean(km)**	**Median(km)**	**(90th)(km)**	**domain(%)**	**Mean(km)**	**Median(km)**	**(90th)(km)**	**domain(%)**
LB	24(38)	20(32)	44(70)	70	24(56)	21(56)	32(81)	95
R_PP	11(17)	1(1)	47(65)	25	10(10)	1(1)	36(36)	0
FBH	31(34)	32(32)	60(67)	26	33(34)	38(38)	57(64)	24
CI	54(70)	55(61)	65(116)	59	53(55)	56(56)	62(67)	44
PMB_2_	47(56)	56(57)	67(91)	41	52(54)	58(58)	61(71)	41
PMB_1_	41(46)	49(49)	66(80)	31	48(50)	57(57)	61(68)	45
WP_2_	35(37)	34(34)	65(71)	20	35(35)	36(36)	59(64)	18
WP_1_	9(9)	0(0)	45(45)	2	25(25)	20(20)	58(58)	6
GI_2_	53(64)	59(62)	66(100)	50	55(57)	60(59)	64(72)	9
GI_1_	54(59)	60(60)	69(88)	36	50(51)	53(53)	65(65)	37
SF	43(45)	49(49)	71(71)	17	40(40)	41(41)	60(60)	0
MB	46(48)	51(51)	73(77)	15	41(41)	43(43)	60(60)	0
PP	20(20)	3(3)	72(71)	5	39(39)	38(38)	65(65)	0
SB	21(21)	7(7)	66(66)	2	29(29)	25(25)	58(58)	0
Average	35(40)	34(36)	62(77)	28	38(41)	39(42)	57(63)	23

Refer to Figure 1b for the release locations. Values in parenthesis extrapolate travel distances of particles that left the domain from the velocity of the last 12 hours before exiting the domain.

**Figure 6:**
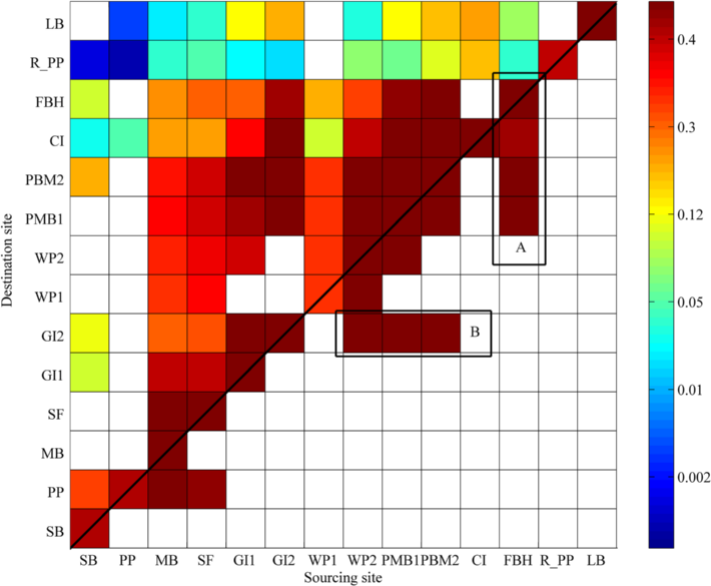
**Connectivity probability matrix: columns are for the release sites, ordered from south to north (left to right) and rows represent the settlement sites from south to north (bottom to top).** The colour scale represents the probability of connectivity between pairs of sites. Overall there is a dominant northward pattern, as reflected by probabilities in the top left half of the matrix. The rectangles represent: **a)** southward transport due to local coastline configuration and resulting circulation pattern, and **b)** westward transport and the importance of westward winds in the morning.

Geographical barriers were found to play an important role on the local circulation patterns, and therefore the fine-scale connectivity patterns, among sites. The hydrodynamic model demonstrated that Garden Island and its connecting bridge to the mainland have isolated the southern seagrass populations (SB and PP) from sites within Cockburn Sound, resulting in greater local connectivity (albeit still weak) with populations towards the south (Figure 4c). The sheltered sites inside Cockburn Sound are thus connected with a gradient of dispersal towards the north. Mangles Bay (MB) located at the lower end of this embayment, is a prime source area; providing fruit to most of these study sites (Figure 4d). The geographical barriers on the coastal circulation at this southern site causes it to not receive fruit from adjacent meadows to the north, i.e. most of its seedlings would likely be self-recruited (Figure 6). The meadows located offshore near the centre of Cockburn Sound, e.g. Parmelia Bank (PMB 1 & 2) and Carnac Island (CI), are more open and more heavily influenced by the stronger and more consistent northward circulation. Dehiscence potential from these sites thus extends to greater distances towards the north (40% up to ~30 km) and there is also substantial east–west transport (~10 km). The central location of these sites also makes them good sink (settlement) sites, i.e., they may readily receive seeds from many sites to the south (Figure 6).

## Discussion

### Dynamics driving dispersal

Within our study area, dispersal distances were greater for offshore meadows, as the fruit were able to be transported in the stronger ocean currents located offshore and were also less likely to be deposited on land when winds switched onshore during the afternoon. Although a northward gradient of potential connectivity is evident, especially when taking into account LDD, within sheltered sites the dispersal processes were heavily influenced by the local hydrodynamics. These fine-scale circulation patterns caused some sites to have a dominant southward transport that opposed the northward wind stresses. This reflects the importance of local water circulation within nearby meadows in the region, which may also influence finer-scale population genetic structure [36]. Coastal landmasses also restricted connectivity among meadows when they affected northward transport; thus, these populations might present differences in terms of shared genotypes, resulting in a subpopulation structure when isolated from the dominant northward transport. Other external factors known to influence dispersal in seagrasses are extreme events such as storms and hurricanes (e.g. [16,37]). Locally, storms typically occur during the winter and lead to strong winds; whereas summer conditions are relatively calmer but with more consistent wind forcing. Therefore, storm effects are not likely to influence average dispersal distance for *P. australis* in this region. However, remote tropical storms as far as ~1000 km from the study area can generate large coastal trapped waves [35,38], which can episodically lead to large cross- and along-shore flows that may also contribute to seed dispersal along this coast.

### Long distance dispersal

This study demonstrates that fruit dispersal in the seagrass *Posidonia australis* is a regional phenomenon. Every year seeds have the capacity to be transported over distances ranging from metres to 100 km or more, supporting the suggestion that in some species of seagrasses, seagrass populations living within these distances should have high demographic connectivity [10]. Dispersal is an essential process in plant population dynamics, where heavy investment in different seed characteristics has improved adaptation to different dispersing agents [39]. In terrestrial environments, this has resulted in specialized mechanisms that are known to efficiently disperse seeds over different spatial scales, depending on the seed characteristics and on the dispersing agents such as wind, water and different animal species (see [40]). These strategies in terrestrial plants have historically been fairly well-studied, although these have mainly focused on average dispersal distances that only imply a typical dispersal scale of a population [41]. A great effort is being put into elucidating long distance dispersal (LDD), connectivity and metapopulation ranges for terrestrial angiosperms (see [42] and references within), which ultimately relates to a population’s survival and evolution [43-45].

The atmospheric and oceanographic conditions in the region result in northward LDD for *P. australis* of at least ~100 km from individual reproductive events. So despite the low probability for reaching these distances, the abundant production of seeds is likely to provide enough recruits to make this an important event over multiple years. This result highlights the importance of reproduction and LDD for this seagrass as well as other seagrasses with floating fruits or propagules [10]. Our estimates of LDD are similar to other seagrass species such as *Zostera marina* that have suggested a dispersal potential of ~100-150 km on rafting shoots bearing seeds (see [26,27]).

### Regional connectivity

On the western coastline of Australia, the climatic and oceanographic context results in a northward trajectory of long distance dispersal. This dependence on wind-generated currents, direct windage, and the location of coastal landmasses that act as barriers to fruit movement, determines contemporary or demographic connectivity and presumably the evolutionary connectivity of populations of *Posidonia australis.* Our simulations suggest important probabilities (~40%) of ‘local’ connectivity (1–15 km) between adjacent meadows, as a result of the exponential decay in the probability of fruit dehiscence over time. This level of dispersal and population connectivity is supported by population genetic evidence (see [46]) that found chaotic genetic patchiness, evidence of active sexually derived recruits, high genetic diversity in local populations and some shared multi-locus genotypes over distances of 8 to 12 kilometres, at the same locations we have modelled. Our results are also supported by observations from other seagrasses. For example, in the tropical seagrass species (*Thalassia testudinum*), Van Dijk et al. [18] estimated dispersal from extrapolation of velocities of tracked floating fruit and inferred distances of up to ~350 km. Their results were also supported by genetic metapopulation distances in the same order [18]. Collectively, these results acknowledge the importance of long distance dispersal to the regional dynamics of seagrass populations with floating fruit or propagules.

There was substantial contemporary dispersal among most populations in this study, clearly suggesting a high level of demographic connectivity among these populations, although this connectivity was generally highly directional towards the north. The exception is the offshore site at Rottnest Island (R_PP), located in a bay protected from the west and facing land to the north. Therefore, this sheltered site acted largely as a sink, opposite to the rest of the sites, as most of the fruit are either self-recruited or entirely lost from the domain; however, there is still some small likelihood that this site receives seeds from most of the southern release sites. Interestingly, this site could present high genetic diversity acquired through a very slow recruiting process from many of the surrounding sites. Successful recruitment from seed dispersal increases gene flow among populations, resulting in greater diversity within local populations, which can also confer resilience to disturbances [47]. Also, high diversity is especially important for threatened ecosystems as it improves adaptation to changing environmental conditions [48]. The ability of marine angiosperms to reproduce vegetatively at high rates, originally led to the idea that seagrasses very seldom recruited from seed. However, observations of greater expansion of meadows than expected from measured clonal growth (e.g. [49]), the high numbers of seeds produced (e.g. [50]), occasional observed recruited seedlings (e.g. [25,51]) and large genetically related metapopulations with high genetic diversity within and among populations (e.g. [52,53]), all suggest that successful seed dispersal events are not rare and continuously contribute to seagrass populations of *P. australis* in southwest Australia.

### Next steps

This exercise allowed us to gain new insight into the range of dispersal that *Posidonia australis* seeds likely experience and thus a more complete picture of connectivity among local populations. Despite every effort put into modelling the dispersal of the fruit as accurate as possible based on the known biology and the physical processes, the size of model domain (~100 km) still constrained estimates of the actual maximum long distance dispersal distances, given that a small percentage of particles left the northern boundary. Thus, even for this relatively large model domain, the connectivity of these local seagrass populations with regions extending further to the north of the study area remains unknown. In addition, our simulations were run for the summer of 2010, and although the general patterns of dispersal are expected to be very similar during other years, the strength of the Leeuwin Current does display some inter-annual variability (i.e. due to the El Niño - La Niña cycle) [34], and hence the overall dispersal distances could vary slightly between years. As with every modelling simulation, there is also always scope to include additional processes, and while we are quite confident that the results of this buoyant transport phase are realistic, for future work we would like to incorporate secondary transport of the seeds at a finer resolution to better understand the dynamics that directly affect post settlement. This study presents the first estimates of local population connectivity through sexual reproduction; however, it does not account for successful recruitment into the reproductive adult population. Further research is also needed to better understand the factors that contribute to successful recruitment in the region, which would provide a comprehensive picture of the full life cycle of this important seagrass species, from reproduction, to seed dispersal, to recruitment, and finally to the successful establishment of new meadows.

## Conclusions

Population dynamics in seagrasses result from a complex balance between vegetative expansion and seed recruitment. Our capacity to infer population dynamics through sexual recruitment depends on our ability to first understand dispersal. In this study we show the strong influence of local wind over the movement of *P. australis* floating fruit*.* Additionally, this coupling with regional to fine scale hydrodynamic processes highlights the importance of local circulation in places where complex water transport is present, such as very shallow coastal areas or semi-enclosed embayments. For surface driven transport, dispersal distances on the order of a few km’s to ~100 km or more are generally predicted, with large distances reached over exposed areas with directionally defined flows. These distances are likely to result in a well-connected south to north corridor along the dominant direction of dispersal. Ultimately an understanding of the dispersal process together with the conditions that favor recruitment and seedling survival can provide us with better strategies to understand and manage these threatened ecosystems.

## Methods

### Hydrodynamic model setup

A numerical circulation model MIKE3 [54] was used to solve the 3D incompressible Reynolds averaged Navier–Stokes equations and was applied to simulate the circulation dynamics in the coastal waters of Perth, Western Australia. The model domain extended ~100 km along the coast with the northern boundary located near Two Rocks (−31.49° S), the southern boundary near Mandurah (−32.53° S), and the western boundary located roughly 40 km offshore where the depth reached just over 100 m (Figure 1a). The size of the domain was chosen as the maximum area allowed by our computational power without compromising resolution for the small scale processes. The study region also included three major islands: Rottnest, Garden and Carnac. This model domain was gridded with an unstructured triangular mesh (~50000 elements), with the grid resolution increasing roughly proportional to the local depth in order to keep the local barotropic Courant numbers less than 0.8 for the typical barotropic time step (~20 s) used in the simulations. The typical (average) grid cell resolution was thus ~140 m in shallow coastal areas (depths <20 m). In the vertical, 10 sigma (terrain-following) layers were used and were distributed uniformly throughout the water column.

A series of hindcast simulations were conducted to validate the hydrodynamic model predictions, which focused specifically on a ~2 month period during the austral summer (November-December) when the fruits of *Posidonia* in Perth’s coastal waters are released [4] and when hydrodynamic data from an extensive field experiment in the region were also available [35]. In this experiment, currents were measured at 8 stations throughout the study area (Figure 1a) and included 5 acoustic Doppler current profilers (ADCPs) that recorded current profiles at the deeper sites (>10 m) and 3 acoustic Doppler velocimeters (ADVs) that recorded currents at a fixed height above the bed at the shallow inshore sites [for details of the instruments and their configurations refer to 35]. We note that the results from this experiment showed that the circulation on both the inner shelf and the Perth coastal lagoon region further inshore (depths <100 m) were not significantly influenced by local stratification (i.e. buoyancy forcing contributions to the circulation were negligible); as a result, density gradients were ignored in the model configuration.

The three open boundaries were forced using a linear interpolation of hourly water levels predicted by the Global Tide Model Data based on TOPEX/POSEIDON altimetry [55]. The water level boundary conditions were also modified to include the typical magnitude of the southward-directed alongshore pressure gradient of 2x10^−7^ m that drives the offshore Leeuwin Current following Godfrey and Ridgway [56] and Smith et al. [57]. Surface wind forcing in the model was based on observations recorded at a local weather station (available every minute from Rottnest Island near the centre of the model domain) by the Bureau of Meteorology and was applied uniformly across the domain using surface drag coefficients from Smith and Banke [58]. The bottom stresses were computed in the model assuming a logarithmic profile in the bottom boundary layer with a hydraulic bottom roughness length scale of *z*_*o*_ *=* 0.01 m (the appropriateness of this roughness value was also investigated by initially conducting a series of sensitivity tests of the influence of bottom roughness on the model results – see below). Horizontal diffusion of momentum was modelled using a Smagorinsky formulation with an eddy viscosity of 0.55 m^2^ s^−1^ and a *k-*ε turbulence closure scheme in the vertical. Finally, Coriolis forcing was included in the model and allowed to spatially vary across the domain. The reader is encouraged to see the software log for one of these simulations presented as an Additional file 1.

Both the field data and model output were interpolated onto a common hourly time base. The data was then compared with a quantitative measure of model ‘*Skill*’, e.g. Warner et al. [59]:

1 $$ \mathit{\mathsf{Skill}}=\mathsf{1}-\frac{{\displaystyle \sum {\left|\mathit{\mathsf{X}}\mathsf{mod}\mathrm{e}\mathsf{l}-\mathit{\mathsf{X}}\mathsf{o}\mathsf{b}\mathsf{s}\right|}^{\mathsf{2}}}}{{\displaystyle \sum {\left(\left|\mathit{\mathsf{X}}\mathsf{mod}\mathrm{e}\mathsf{l}-\overline{\mathit{\mathsf{X}}\mathsf{o}\mathsf{b}\mathsf{s}}\right|+\left|\mathit{\mathsf{X}}\mathsf{o}\mathsf{b}\mathsf{s}-\overline{\mathit{\mathsf{X}}\mathsf{o}\mathsf{b}\mathsf{s}}\right|\right)}^{\mathsf{2}}}} $$

where *X* represents the variable to be analysed, either as predicted by the model (*X*_model_) or the observations (*X*_obs_), and where the overbars denote time-averaged values. For Eq. (1), perfect model-data agreement thus results in value of one whereas a value of zero implies complete disagreement. The *Skill* was computed at each of the observation sites and was applied by separately comparing both the depth-averaged flows (*U*_*d*_,*V*_*d*_), as well as the surface flows (*U*_*s*_,*V*_*s*_) at the deeper sites (Figure 1a). We note that we focus on evaluating the performance of the model to predict the subtidal component of the circulation since this had by far the greatest influence on the net transport of seeds fruit the five day dehiscence wind period. Given the weak oscillatory tidal currents in Perth’s coastal waters [35], the maximum tidal excursion lengths are typically <500 m. Nevertheless, in all of the particle dispersal simulations, tidal currents were still included. In addition, water levels computed from the ADCP and ADV bottom-mounted pressure sensors corrected for local atmospheric pressure, were also compared to the model output. To assess the current variability at each site, for both the field data and model predictions, a principal component analysis was performed on the variance of the easterly (*U*) and northerly (*V*) velocity components [60]. The time series of both the observed and modelled currents were then rotated into the major and minor axes of the variance; in most cases these defined the alongshore and cross-shore flow directions, respectively (see below).

### Fruit particle tracking (dispersal and connectivity simulations)

Output from the hydrodynamic transport model was used to drive a Lagrangian particle tracking model that advected particles with the fruit characteristics and simulated dispersal as a random walk process. In the random walk, horizontal dispersion coefficients were based on a scaled horizontal eddy viscosity from the Smagorinsky formulation (a typical value of 0.01 was used for the scaling factor, although the results were advection-dominated and negligibly influenced when this parameter was varied over more than an order of magnitude). For this application the dispersal of *P. australis* floating fruit were treated as passive particles transported at the sea surface. In addition to the transport of particles by the ocean surface current vectors from the hydrodynamic model, the additional transport by windage on the floating *P. australis* fruit, as detailed in Ruiz-Montoya et al. [4], were included in the model. The windage consisted of 1.2% of the local wind speed and was added to the surface transport, which was based on field studies of the response of tracked *P. australis* fruit to different wind forcing conditions (refer to [4]). These coupled hydrodynamic-particle tracking simulations focused specifically on the period from the 24th of November to the 15th December 2010, when local *P. australis* fruit were observed to be released in the coastal waters of Perth (see the Additional file 1 for the model settings). Fruit release in the region occurs annually and the environmental regime is quite consistent with easterly winds during the morning and strong seabreezes in the afternoon [35,61]. The transport of fruit was assessed from fourteen meadows chosen for fruit release (Figure 1b). These sites spanned the full study area; however there were more sites within Cockburn Sound, firstly because these populations are among the best mapped along Perth [62], and secondly because most of these sites coincided with genetic sampling studies by Sinclair et al. [46,63] that can be utilised in future work. Each release site consisted of 30 by 30 m zones over which the seagrass fruit were randomly released. Twenty five particles per site were released every hour during the study period (~12,500 per site), which is likely an under estimate of the seeds produced and released in these areas by a factor of ~4 (M. Waycott, personal communication). This maximum number of simulated particles was based on the computational limitations of the particle tracking; however, they will have a negligible influence on the actual probability distributions of dispersal that are the focus here. Each particle (fruit) was allowed to travel for up to 5 days (based on results from [4]). Any particles that left the model domain during this period were recorded as lost; however an extrapolation on the velocity of the last 12 hours before leaving the domain was applied for the remaining time until final dehiscence, to investigate LDD potential. From Ruiz-Montoya et al. [4] where ~1200 fruit were monitored, the percent of remaining (non-dehisced) fruit were empirically fit to *y* = *a* exp^(−*bt*)^, where *t* represents time in days, *y* the dehiscence percentage of the studied sample, which gave *a =* 117 and *b =* 0.78 d^−1^ (see [4]). This expression represents an exponential distribution of a continuous random variable; hence its probability density function (PDF) of dehiscence was defined as *P*_(t)_ = 0.781exp^(−0.781t)^. After each particle tracking simulation, the individual particle trajectories were analysed based on their positions every second hour. Using the dehiscence probability *P*_(*t*)_ curve, the settlement locations of individual seeds were then recorded. Horizontal transport during the rapid settlement phase was not incorporated, due to the fast settling velocity of *P. australis* seeds; for example, a transport of only 15 m would result in a maximum depth of 20 m from a current speed of 0.075 m s^−1^, which is considered relatively fast in the study area (see [35]). The probabilities for settlement were then spatially assigned among a 500 m × 500 m grid of the entire study area, thereby producing a settlement probability map. This process was carried out for the 14 different release sites. Finally, this information was used to quantify the connectivity between the different sample sites by computing a connectivity matrix for the region, where the rows represent the meadow receiving the seed and intersect columns that represent the meadow sourcing the fruit [1].

## Additional file

Additional file 1: **Supplementary Material.**
